# Cambio climático y salud: un imperativo de vigilancia y acción desde el sector salud

**DOI:** 10.7705/biomedica.8232

**Published:** 2025-11-27

**Authors:** Luis Jorge Hernández-Flórez

**Affiliations:** 1 Facultad de Medicina, Universidad de los Andes, Bogotá D. C. , Colombia Universidad de los Andes Facultad de Medicina Universidad de los Andes Bogotá D. C. Colombia

El cambio climático se ha consolidado como el mayor desafío sanitario del siglo XXI. Sus efectos sobre la salud humana no son hipotéticos: se expresan ya en el incremento de las olas de calor, en la intensificación de los fenómenos hidrometeorológicos extremos y en la modificación de los ecosistemas que sostienen la vida. El sector salud está llamado, no solo a responder a las consecuencias inmediatas, sino a anticiparse y generar evidencia que oriente acciones de mitigación y adaptación.

La relación entre variabilidad climática, cambio climático y salud se manifiesta en múltiples dimensiones. En primer lugar, las enfermedades transmisibles constituyen un frente crítico: la expansión de vectores como *Aedes aegypti* y *Anopheles* spp. está ligada al aumento de la temperatura y a cambios en la pluviosidad, lo cual favorece la transmisión de malaria, dengue, fiebre de chikunguña y enfermedad por el virus del Zika, así como la emergencia y reemergencia de agentes patógenos. A ello, se suma la amenaza de enfermedades zoonóticas, recordando que la mayoría de los agentes infecciosos tienen origen animal. Este panorama obliga a reforzar las coberturas de vacunación, hoy insuficientes, e incorporar con mayor fuerza el enfoque de *One Health*, que articula la salud humana, la animal y la ambiental.

En segundo lugar, el cambio climático incide en las enfermedades crónicas no transmisibles. El incremento de la temperatura y la contaminación atmosférica elevan la carga de enfermedades cardiovasculares, cerebrovasculares y respiratorias; la exposición prolongada a contaminantes ambientales está asociada con el cáncer y con los trastornos metabólicos; y el estrés climático y social se traducen en un aumento sostenido de las enfermedades mentales. Se trata de una serie de impactos continuos que exigen fortalecer la promoción de la salud, la vigilancia epidemiológica ambiental y la investigación interdisciplinaria.

Un tercer ámbito lo constituyen el complejo trauma-violencia, las emergencias y los desastres. Las inundaciones, sequías y deslizamientos, cada vez más frecuentes, generan pérdidas humanas, desplazamientos poblacionales, inseguridad alimentaria y brotes epidémicos. Estos eventos, además de exponer la fragilidad de la infraestructura sanitaria, intensifican la conflictividad social y la violencia, profundizando inequidades preexistentes.

En Colombia, la Ley 1931 de 2018 estableció los *Planes integrales de gestión del cambio climático territoriales* como instrumentos centrales para enfrentar esta crisis. El reto es articularlos con los planes territoriales de salud, incorporando intervenciones colectivas e individuales de monitoreo y adaptación. De esta manera, el sector salud se posiciona como actor estratégico para garantizar la resiliencia de las comunidades y la sostenibilidad de los sistemas de vida.

La evidencia científica, incluida la presentada en este número de *Biomédica*, debe traducirse en acción política. La mitigación y adaptación al cambio climático no son opciones, son imperativos éticos y de supervivencia. Desde la salud pública, reforzar la vigilancia, la investigación y la integración intersectorial, son tareas urgentes para proteger la vida en todas sus formas.

